# Isolated Unilateral Total Absence of the Pectoralis Minor Muscle: A Rare Finding in Two Cases

**DOI:** 10.7759/cureus.64421

**Published:** 2024-07-12

**Authors:** Anasuya Ghosh, Subhramoy Chaudhury, Grace Suganya

**Affiliations:** 1 Anatomy, All India Institute of Medical Sciences, Kalyani, Kalyani, IND; 2 Anatomy, Santiniketan Medical College, Kolkata, IND

**Keywords:** pectoralis minor muscle, sporadic variant, reconstruction, chest wall abnormality, poland syndrome

## Abstract

Anatomical variations are observed at times during a routine dissection process and some of them are clinically relevant as they can lead to certain clinical presentations or situations that are difficult to anticipate without the knowledge about their possibility. The unilateral non-syndromic complete absence of pectoral muscles is very rare. Their absence is always found to be associated with syndromes like Poland syndrome or Sprengel’s deformity. During the routine anatomical dissection, we encountered two cases of non-syndromic complete unilateral absence of the pectoralis minor muscle. On further inspection of the cadaver in both the cases, no other bony (ribs, scapula), vascular, breast abnormalities, or muscular aplasia (fibers of the serratus anterior or pectoralis major) was noted. As the pectoralis minor muscle serves as the potential surgical landmark and can also be used as the myo-cutaneous flaps for facial reanimation surgeries and in thumb opponensplasty, the absence of the pectoralis minor muscle would come as a surprise for the surgeons during the process of harvesting the flap for these procedures, so the possibility of this kind of rare variation should be documented.

## Introduction

The thoracic cage is augmented by thoracic wall muscles like the pectoralis major muscle, pectoralis minor muscle, serratus anterior, intercostal muscles, levator scapulae, and subclavius [1]. The underdevelopment or absence of these muscles is always associated with syndromes like Poland and Sprengel’s deformity. Though the absence of pectoral muscles accounts for about 1 in 11000, the literature reporting isolated, unilateral, complete absence of the pectoralis minor muscle is very rare [2]. We would like to report two cases of non-syndromic complete unilateral absence of the pectoralis minor muscle. One interesting thing was that the two cases were found in two different regions of the world. The first case was seen during dissection in AIIMS, Kalyani, West Bengal, India, and the second case was found during dissection at the wet cadaver lab at the Medical University of the Americas, Charlestown, St Kitts and Nevis. This would help the clinicians do proper screening to find all other abnormalities associated with such conditions so as to prevent further complications.

## Case presentation

Case 1

**Figure 1:**
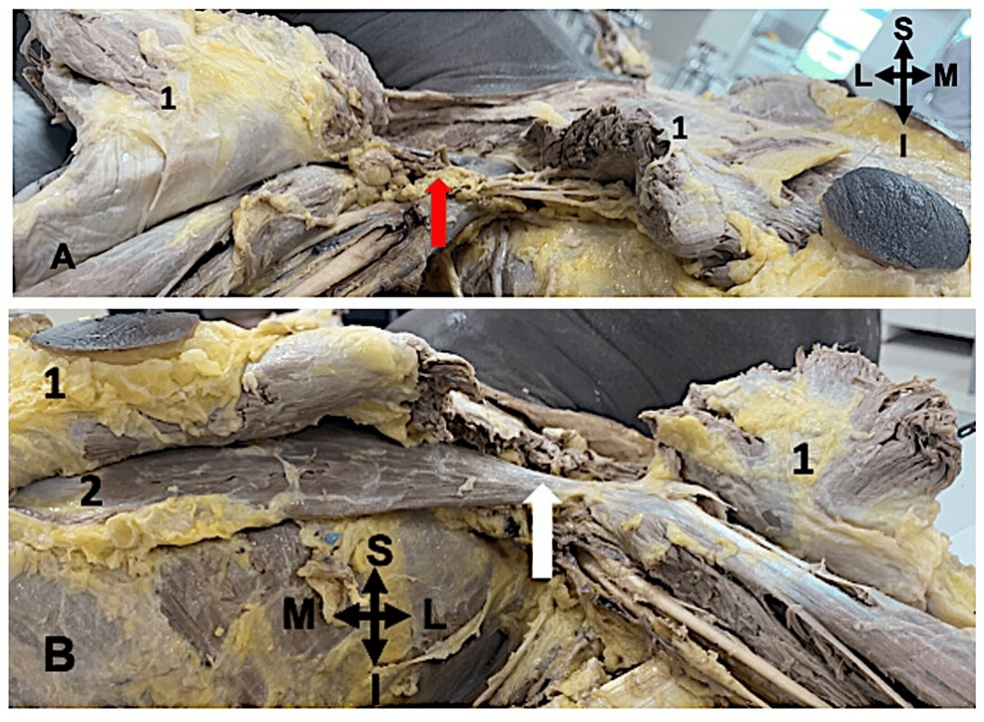
The cadaveric image showing the absence of the pectoralis minor muscle in case 1 1: Pectoralis major muscle; 2: Pectoralis minor muscle; A: Right side; B: Left side; S: Superior; I: Inferior; L: Lateral; M: Medial The red arrow indicates the absence of the pectoralis minor muscle on the right side and the white arrow indicates the presence of the pectoralis minor muscle on the left side

A case of a complete absence of the pectoralis minor muscle on the right side of a cadaver was found while doing routine dissections by first-year medical students. It was an elderly male cadaver, aged around 78 years, during February 2023. Before dissection, no gross asymmetry was noted on the anterior chest wall. On further inspection of the cadaver, no other bony (ribs, scapula, phalanges of the upper limb), vascular, breast abnormalities, or muscular aplasia (fibers of the serratus anterior or pectoralis major muscle) was noted. The digits of both hands were symmetrical without any visible anomaly. The axillary vessels and nerves from the brachial plexus on the right side were covered by an axillary sheath and connective tissue directly below the fibers of the pectoralis major muscle. In the present case, no family history was available indicating any upper limb defects. Both the pectoral nerves were supplying the pectoralis major muscle. The clavipectoral fascia was present below the pectoralis major muscle which extended above covering the subclavius. During further routine dissection of the thorax, no malformations were detected involving the thoracic cage, heart, lung, etc. The cadaveric image showing the absence of the pectoralis minor muscle is depicted in Figure 1.

Case 2

**Figure 2:**
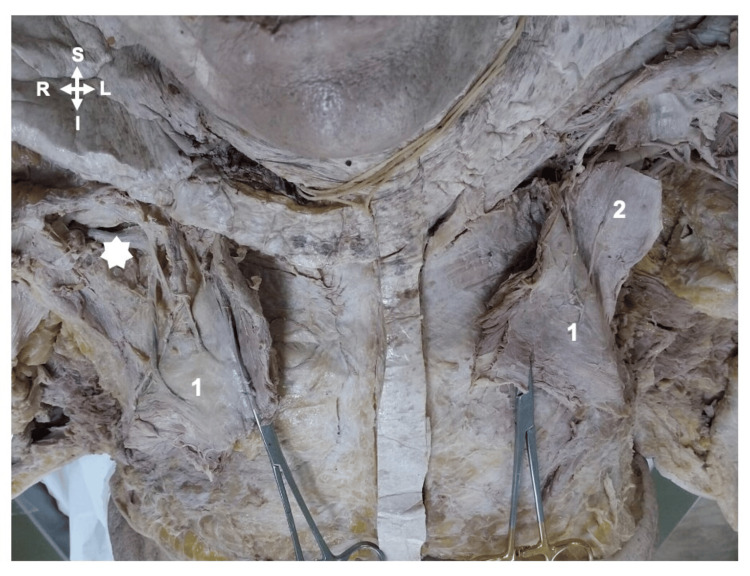
The cadaveric image showing the absence of pectoralis minor muscle in case 2 1: Pectoralis major muscle; 2: Pectoralis minor muscle; S: Superior; I: Inferior; R: Right; L: Left White star showing the absence of the pectoralis minor muscle on the right side

A case of isolated, unilateral, complete absence of the pectoralis minor muscle on the right side was found during routine dissection classes at the Wet Cadaver Lab in March 2018. The cadaver was an obese male, aged around 65 years. The pectoralis major muscle was normal and present on both sides; the pectoralis minor muscle was intact and normal on the left side. On the right side, the pectoralis minor muscle was absent, the axillary sheath containing vessels and nerves was covered by a thick connective tissue layer deep into the pectoralis major muscle. No other muscular (pectoralis major or serratus anterior) or bony anomalies (scapula or ribs or phalanges of the upper limb), underdeveloped breast, or anomalies related to viscera like heart or kidney were noted. There was no family history that pointed to anomalies of the upper limbs or chest wall. The cadaveric image showing the absence of the pectoralis minor muscle is depicted in Figure 2.

## Discussion

Two cases of isolated unilateral complete absence of pectoralis minor muscle were detected during routine cadaveric dissection by the authors at two different places.

Poland syndrome is a rare birth defect associated with chest wall abnormalities like partial or complete agenesis of muscles like pectoralis major muscle, pectoralis minor muscle, serratus anterior, and latissimus dorsi, and bony deformations like underdeveloped ribs and scapula [3,4]. Very rarely, it also presents with underdeveloped kidneys, cardiac dextroposition, problems with hemostasis, malignancies like lymphoma, leukemia, leiomyosarcoma, breast carcinoma, and anomalies of the upper limb [4,5]. Though mostly the etiology of Poland syndrome is not known, its sporadic variant is encountered more than the familial variant [4]. The incidence varies from 1 in 20,000 to 1 in 50,000 live births. It is three times more common in males than females and involves the right side (75%) more than the left side [5]. 

Stylianos et al. and Bainbridge et al. using computed tomography (CT) documented the lack of pectoral muscles in ten and four cases of Poland syndrome respectively [1,6]. In both the studies, either both pectoralis major and minor muscles were absent, or if only the pectoralis minor muscle was absent, it was associated with other upper limb abnormalities. An isolated absence of the pectoralis minor muscle was not found. So, the findings of both of these studies were not consistent with our report.

However, an isolated absence of the pectoralis minor muscle has been reported by Gardiner et al. as an incidental finding in a five-year-old child during a facial reanimation procedure. There were no other gross chest wall abnormalities or features suggestive of syndromes present [7] which is consistent with the present study.

The absence of muscle may be due to failure of development of the muscle itself, or the developed muscular rudiments might undergo degeneration because of a lack of attachment to bones, or due to the exposure to ergot alkaloids in the first trimester of pregnancy, or it may be due to the upheaval of the lateral plate mesoderm at 4 to 5 gestational weeks [3,4]. Across the globe, though there were various theories postulated for the absence of pectoral muscles, the problem that was encountered in the separation of the pectoral mesenchyme into the pectoral major and minor anlage at the 6th to 7th week of gestation is considered an acceptable fact. This may be due to the vascular insult during the development of limb buds [2].

The congenital defects in the pectoral muscles get obscured usually as they do not present with any noticeable functional disabilities unless associated with syndromes [7]. To rule out the association between leukemia and anomalies of the urogenital system and the absence of a pectoral group of muscles, a complete hematological test, and an ultrasound are recommended, even though the patient is asymptomatic [7]. Some rare associations of Poland syndrome with facial nerve palsy [8] and carcinoma of the breast were also reported [5]. Apart from that pectoralis minor muscle is solely used as the potential myocutaneous flap in case of facial reanimation surgery and thumb opponesplasty [7,9]. Though the isolated absence of the pectoralis minor muscle is an incidental finding, it is imperative for the clinician to exclude the other syndromes associated with it.

Sprengel’s deformity is another condition that is associated with the agenesis of the pectoral group of muscles and the webbing of fingers [7]. Another main cosmetic problem with these syndromes is the deficiency of the axillary fold in the anterior aspect [6]. Bainbridge et al., when trying to correct this deformity in the axillary fold with the pedicle from the latissimus dorsi, found that the vascularity of the pedicle was completely absent in the lower half of the muscle flap. Thus, CT is warranted preoperatively to evaluate the level of muscular disability in and around the chest wall for a good reconstruction [8]. Thus, the varied spectrum of associations between certain other conditions like facial nerve palsy, breast carcinoma, leukemia, and renal abnormalities with the Poland syndrome should be kept in mind by the clinician while encountering these cases, even if they are incidental findings. 

## Conclusions

In our report, both cases are male with an absent pectoralis minor muscle on the right side. Although the absence of other muscular or vascular chest wall abnormalities and upper limb defects prevent us from labeling this as a case of Poland syndrome, still, the sporadic variants of Poland syndrome are more common in the male population, and the prevalence on the right side is observed three times more frequently than the left side. So, both of these cases could be regarded as a sporadic variant of Poland’s syndrome or isolated cases of non-syndromic, congenital, complete absence of the pectoralis minor muscle unilaterally. Since flap from the pectoralis minor muscle is used in facial reanimation surgeries and opponensplasty of thumb, their absence should always be kept in mind by surgeons preoperatively to prevent encountering unexpected variations during surgical procedures. Thus, the present case report would help the clinicians and surgeons for proper evaluation of the cases and prevent complications.
